# Prevention of Pneumonia

**Published:** 1918-11

**Authors:** 


***Prevention of Pneumonia.*** By RUFUS COLE. *Journal of the American Medical Association*, August 24, 1918.

Today in considering this question, not only must we consider acute lobar pneumonia, but we must also include in our discussion broncho or lobular pneumonia having an entirely different etiology.

Both kinds of pneumonia have prevailed in most of the camps, frequently simultaneously.

Our consideration of the prevention of pneumonia will probably be rendered more clear by an independent consideration of the two diseases, for they must be considered such, at least from the standpoint of prevention.

*Acute Lobar Pneumonia due to Pneumococcus.* The pneumococcus is a widely distributed organism in the mouths of healthy individuals, and since it survives for a considerable length of time in dust, great opportunities exist for its wide-spread distribution. A demonstration that pneumococci are not all identical, that the types responsible for two-thirds of the cases, and these the most severe, are found only in the mouths of those sick with the disease, in the dust of their immediate environment, and in the mouths of a very limited number of carriers, at once justifies attempts to prevent pneumonia by limiting the distribution of these more parasitic types of pneumococci. In pneumonia, due to these types of pneumococci, direct infection of one patient from another is not obvious nearly so often as it is in the case of diphtheria, for instance. In pneumonia, examples have been seen of contact infection, and recent studies have demonstrated the not infrequent association due to pneumococci of the same type. This suffices to justify the attempts to prevent the spread of infection from patients sick with these types of pneumonia.

If pneumococci from patients suffering from pneumonia are taken into the respiratory tract of healthy individuals, they are more likely to produce severe infections than are organisms that have been leading a more or less saprophytic existence. Therefore, stress should be laid on preventing the dissemination of pneumococci from patients sick with the disease.

*Isolation and Vaccination.* Lowered resistance cannot be neglected. In the prevention of pneumonia, measures to prevent undue exposure and lowering of resistance should be instituted, and isolation of pneumonia patients should be carried out to as great an extent as is practicable.

While a considerable number of cases of pneumonia have occurred among the uninoculated men, not a case of pneumonia at Camp Upton, due to pneumococci types I, II or III, has occurred among the inoculated.

*Streptococcus Pneumonia.* So far as can be determined, bronchopneumonia which has existed so widely in the camps has been due to one organism, a hemolytic streptococcus. All the streptococci isolated have not been identical in their power to ferment the various sugars, and they have differed to a less degree in the appearance of the colonies formed by them. It is not yet known whether all these organisms are immunologically identical. If they are not identical, we must consider that the essential factor in inducing the epidemic was something apart from the organisms themselves.

It may be stated that actively hemolytic streptococci are found only with the greatest rarity in normal throats, except in the presence of epidemics of the so-called streptococcus sore throat or streptococcus pneumonia.

At present we seem justified in considering persons who harbor large numbers of actively hemolytic streptococci in the throat as potential carriers of this disease. These human streptococci are tenacious of life, resistant to drying, and other deleterious influences. The possibility of infection through food should not be disregarded.

Our present knowledge indicates that the infectious agent is spread by fairly direct transfer of the infectious agent from the patient sick with the disease, or from healthy carriers, either by droplet infection or by the bacteria carried in particles of dust. The pathology of the disease suggests that the organisms are not highly invasive for man, as they take root and grow in man with difficulty, and even when once established they do not intensively invade the human tissues. They are highly " fatal " to man, but not extremely " virulent " for man.

*Epidemics of Measles.* This disease seems to render the respiratory mucous membrane especially susceptible for secondary infection, particulary with the streptoccus. While on admission to the measles wards only about 11 o/o of the throats showed the presence of hemolytic streptococci, after the patients had been in the wards from 8 to 14 days, between 50 o/o and 60 o/o harbored these organisms in their throats.

It seems probable that without the extensive occurrence of measles the streptococcus pulmonary infections would never have reached the widespread prevalence that they attained. The author cannot help suggesting that the epidemic started as a streptococcus infection in the respiratory mucous membrane of measles patients. The streptococci rapidly increasing in virulence were then able to attack patients suffering from other respiratory diseases, such as lobar pneumonia. And finally, the streptococci have acquired such virulence that now even healthy persons may be infected.

*Measures to Prevent Infection.* The efforts to prevent and limit this disease should consist first in the institution of special precautionary measures to prevent the infection of those persons in whom a high degree of susceptibility exists, especially patients with measles, lobar pneumonia and other respiratory infections; second, since the streptococci are present in greatest numbers and are most virulent in patients already suffering from these diseases, in endeavoring to obtain an etiologic diagnosis in these patients at the earliest possible moment, and in isolating them as rigidly as possible. The third measure, which would be of the greatest value, is preventive inoculation. In the case of streptococcus, this is not so simple as it might appear at first sight, for little is known concerning immunity to streptococci. Finally, the detection and isolation of healthy carriers, while theoretically of great importance, seem at present impossible and impracticable tasks. Protection of the soldier from influences which may lower his general resistance is also undoubtedly of importance.

Emphasis should be laid on the matter of early diagnosis, and especially the matter of proper isolation of cases of infectious disease. For these measures to be carried out, there are three essentials : first, knowledge by the surgeons of the early signs and symptoms in those diseases in which diagnosis is made by these features alone, notably the exanthemata; second, well equipped laboratories, in which etiologic diagnoses may be properly made; third, properly organized infectious disease hospitals.

The personnel of the infectious disease hospital should be distinct from that of the general hospital. So far as possible patients suffering from pneumonia due to the different types of pneumococci should be treated in different wards. Patients suffering from streptococcus infections should be rigidly isolated. Measles and pneumonia patients having hemolytic streptococci in their throats should not be placed in the same wards or rooms with those not carrying such organisms.

In one camp where the measles cases carrying streptococci had been carefully isolated from the so-called "clean measles cases" complications among the latter have been practically eliminated.

